# Guillain-Barré Syndrome Following Hand, Foot, and Mouth Disease in an Adult Patient

**DOI:** 10.7759/cureus.45423

**Published:** 2023-09-17

**Authors:** André Teixeira, Diana Torres Lima, Ana Almeida Pereira, Marta Amaral-Silva, Ana Catarina Miguéns

**Affiliations:** 1 Physical Medicine and Rehabilitation, Centro Hospitalar Universitário Lisboa Central, Lisbon, PRT

**Keywords:** acute inflammatory demyelinating polyneuropathy (aidp), neurological rehabilitation, coxsackievirus, hand-foot-mouth disease, guillain-barré syndrome

## Abstract

Guillain-Barré syndrome (GBS) stands as one of the primary causes of acute flaccid paralysis. It includes acute-onset peripheral nerve lesions and typically follows a monophasic course. Its etiopathogenesis is linked to an immune-mediated response to a prior infection, often respiratory or intestinal. The main variants of GBS are acute inflammatory demyelinating polyneuropathy, which accounts for approximately 90% of cases in the USA and Europe, and acute motor axonal neuropathy, responsible for about 10% of cases in the USA and Europe. From the literature review, only one case of GBS preceded by hand, foot, and mouth disease (HFMD) has been described. The authors report a rare clinical case of typical GBS after HFMD. Recognizing this adult-onset disease as a potential preceding infection of GBS is crucial for early diagnosis and treatment. Additionally, the integration into a rehabilitation program adjusted to the deficits plays an important role in motor and functional recovery.

## Introduction

Guillain-Barré syndrome (GBS) is the most common cause of acute flaccid paralysis worldwide. It is defined as an immune-mediated inflammatory demyelinating polyradiculoneuropathy in 90% of cases. The estimated global incidence is 0.6-4/100,000. It is preceded in more than two-thirds of cases by a respiratory or gastrointestinal infection and less commonly after surgery or vaccination [1]. Among the various microorganisms with established associations, *Campylobacter jejuni* is the most prevalent, accounting for one-third of cases, and less frequently *Borrelia* and *Mycoplasma pneumoniae*. Viruses such as echovirus, coxsackievirus, varicella-zoster virus, paramyxovirus, rubella virus, influenza virus, HIV, and more recentlyZika and SARS-CoV-2 have also been associated [1]. Coxsackieviruses, often associated with hand, foot, and mouth disease (HFMD), meningitis, and myocarditis, are an identified cause of GBS, albeit extremely rare, as described in the literature (only one case published in 2000 by Mori et al.) [2]. HFMD primarily affects children, and the diagnosis is clinical with the appearance of a painful oral enanthem and rash on the palmar and plantar surfaces. It can, r*arely*, affect adults, with most cases being asymptomatic. The most frequently implicated agent is coxsackievirus A16, although other coxsackie or enteroviruses may be involved [3]. GBS typically presents with distal-proximal sensory changes progression, progressive ascending and symmetric muscle weakness (paraparesis/tetraparesis), and absence of deep tendon reflexes. Cranial nerve involvement occurs in approximately 45-75% of cases (mostly after limb involvement), and about 40% of cases are associated with respiratory and oropharyngeal muscle weakness. The clinical presentation occurs days to weeks after the preceding event and reaches a plateau phase within four weeks. The definitive diagnosis is confirmed by neurophysiological studies and cerebrospinal fluid analysis with albuminocytological dissociation, which represents a total increase in proteins, particularly albumin, without pleocytosis. Disease-modifying treatment includes intravenous immunoglobulin and/or plasmapheresis [4]. Early assessment and implementation of a multimodal rehabilitation program play a crucial role in preventing complications, promoting deficit regression, and minimizing disability [5]. Prognosis, although favorable, depends on early diagnosis, access to disease-modifying treatments, and supportive care. Advanced age, *C. jejuni* infection, the need for ventilation, axonal variant, duration of the plateau phase, and motor deficit at the peak of paralysis have been identified as predictors of poor prognosis. In this article, we present a rare case of GBS following HFMD in an immunocompetent adult.

## Case presentation

**Figure 1:**
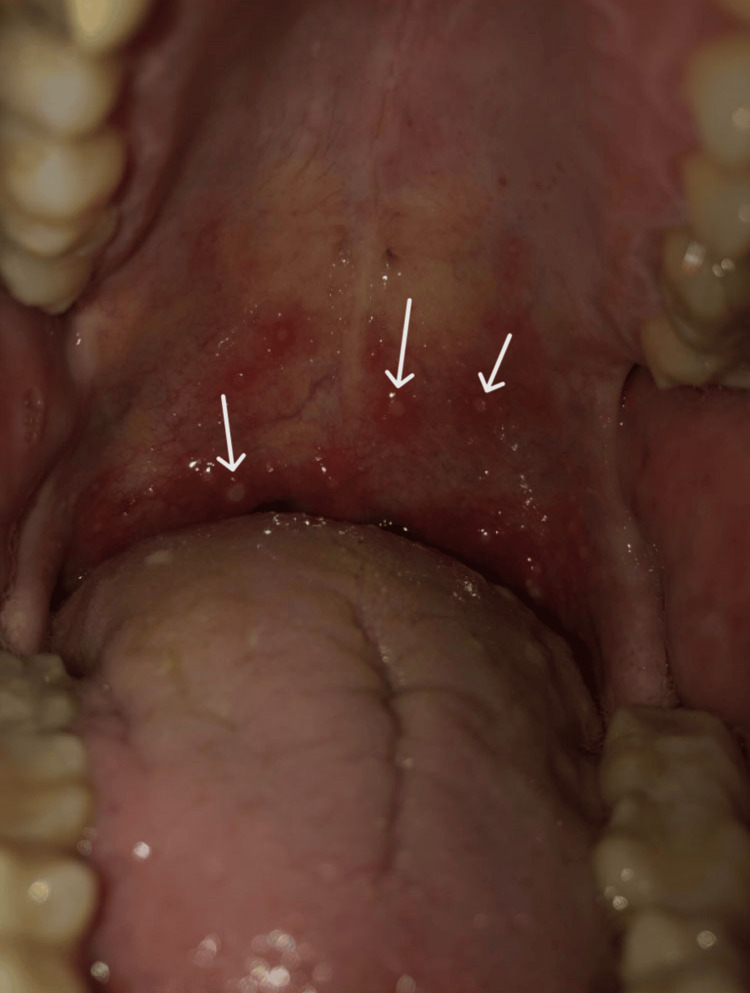
Oral enanthem located on the soft palate (arrows).

A previously healthy 45-year-old Caucasian man was admitted to the emergency department with sudden onset symmetrical paresthesias in a stocking-and-glove distribution and muscle weakness in the lower limbs with progressive worsening over the last 24 hours. Upon admission, asymmetric proximal predominant tetraparesis, hypoesthesia to temperature, and pain in a stocking-and-glove pattern, as well as impaired vibratory sensation in the lower limbs were noted. Deep tendon reflexes were absent. Due to suspicion of GBS, he was admitted to the Intensive Care Unit for close monitoring due to the risk of clinical progression with ventilatory compromise and the need for assisted ventilation. Comprehensive diagnostic workup included laboratory testing, lumbar puncture, and neurophysiological studies. Notable findings included positive IgG antibodies to the Hepatitis A virus and positive IgG antibodies to the Epstein-Barr virus nuclear antigen. Cerebrospinal fluid analysis revealed albuminocytological dissociation, and electromyography showed predominantly demyelinating sensory-motor abnormalities, especially in the upper limbs, confirming the initially suspected diagnosis. Intravenous immunoglobulin was initiated at 400 mg/kg/day for five days. After a multidisciplinary discussion, it was followed by seven sessions of plasmapheresis due to clinical worsening with global respiratory failure and the need for invasive mechanical ventilation on the fifth day of hospitalization. He was successfully extubated after 10 days without complications. At four weeks, he reached a plateau phase, presenting with dysphonia, right peripheral facial paresis, predominantly proximal tetraparesis (Grade 2 in shoulder abductors and flexors; Grade 3 in elbow flexors and extensors, wrist flexors and extensors, finger flexors and extensors, and thumb movements; Grade 4 in wrist flexors, Grade 2 in all hip movements; and Grade 3 in the other lower limb segments, according to the Medical Research Council muscle scale). He had residual hypoesthesia to temperature and pain in a stocking-and-glove pattern and vibratory sensory loss in the lower limbs, as well as absent biceps, triceps, brachioradialis, patellar, and Achilles tendon reflexes. Given his clinical stability and persistent neuromotor deficits (Table 1) with partial dependency on activities of daily living (ADL), he was transferred to the Physical and Rehabilitation Medicine ward. On admission, the patient required maximum assistance with transfers, bathing, dressing, and feeding, scoring 35/100 on the Barthel scale, with 100 being complete independence, and 67/126 on the Functional Independence Measure (FIM), with 126 being complete independence. He underwent an interdisciplinary rehabilitation program, including speech therapy with the goal of voice rehabilitation and improvement of orofacial motor function, physiotherapy with the aim of maintaining joint mobility, muscle strengthening, sensory re-education, and functional improvement in bed and wheelchair, progressing to balance training in an upright position and walking, and occupational therapy with the goal of muscle strengthening, manual dexterity, sensory re-education, and improvement in ADL performance. Epidemiological investigation revealed a history of fever and oral enanthem in the previous week (Figure 1), temporally associated with caring for his daughter with HFMD. Due to persistent and severe neuropathic pain, pregabalin and duloxetine were initiated and titrated until the nearly complete resolution of symptoms. Pulmonary function testing did not reveal significant ventilatory abnormalities during hospitalization. Throughout his hospital stay, he demonstrated improvement in dysphonia and neuromotor function, ultimately being discharged after eight weeks of hospitalization. His vocal quality remained unchanged, with symmetric facial expression and normal strength in all segments except for right shoulder abduction, left knee flexion, and left ankle dorsiflexion. Deep tendon reflexes remained globally absent, and sensory abnormalities improved, with a reduction in the area of hypoesthesia (Table 1). Functionally, he showed significant improvement, achieving complete independence in ADL and being able to walk independently for long distances, with a Barthel score of 100/100 and FIM score of 126/126.

**Table 1:** Evolution of muscle strength (according to the Medical Research Council muscle scale), sensory examination, and deep tendon reflexes examination on the date of admission and discharge from the Physical and Rehabilitation Medicine ward.

Neurological examination	-	Admission		Discharge	
		Right	Left	Right	Left
Motor examination	Shoulder abduction	G2	G2	G4	G5
	Shoulder flexion	G2	G2	G4	G5
	Elbow flexion	G3	G3	G5	G5
	Elbow extension	G3	G3	G5	G5
	Wrist flexion	G4	G4	G5	G5
	Wrist extension	G3	G3	G5	G5
	Finger flexion	G3	G3	G5	G5
	Finger extension	G3	G3	G4	G5
	Thumb (flexion, extension, abduction adduction, opposition)	G3	G3	G5	G5
	Hip flexion	G2	G2	G5	G5
	Hip extension	G2	G2	G5	G4
	Hip abduction	G2	G2	G5	G5
	Hip adduction	G2	G2	G5	G5
	Knee flexion	G3	G3	G5	G4
	Knee extension	G3+	G3	G5	G5
	Ankle dorsiflexion	G3	G3	G5	G4
	Ankle plantar flexion	G3	G3	G5	G5
Sensory examination	Pin prick	Decreased sensation in a stocking-and-glove distribution extending to the 2/3 of upper and lower limbs		Decreased sensation on the plantar face of the toes	
	Light touch	Decreased sensation in a stocking-and-glove distribution extending to the 2/3 of upper and lower limbs		Decreased sensation on the plantar face of the toes	
	Vibratory sensitivity	Altered in upper and lower limb		Altered in lower limb	
Reflex examination	Biceps	Absent		Absent	
	Triceps				
	Brachioradialis				
	Patellar				
	Achilles				

## Discussion

There are few cases reported in the literature of GBS following an infection with the coxsackievirus family, and only one following HFMD. This can be explained by the fact that HFMD in adults often presents as an asymptomatic and self-limiting infection. Although it was not possible to identify the specific etiological agent, the presence of fever and oral enanthem, in the described epidemiological context, makes this diagnosis highly likely. While the majority of GBS cases have a good functional prognosis, with 77% of patients capable of independent ambulation at six months, there is a significant risk of sequelae affecting function and quality of life [4]. Vital prognosis depends on various factors, with the most critical being the motor deficit at the peak of paralysis, highlighting the importance of monitoring bulbar weakness, respiratory muscle function, and autonomic dysfunction. Despite the presence of predictors of poor prognosis such as motor deficit and the need for ventilation, the patient achieved complete functional recovery at six months, with social and professional reintegration [4]. Although evidence regarding rehabilitation in GBS is lacking, this case demonstrates the benefits of an intensive rehabilitation program [5]. While it is not possible to isolate the benefits of the rehabilitation program from the spontaneous recovery phase, over the eight weeks of hospitalization, the patient showed progressive improvement in overall function.

## Conclusions

The authors aim to raise awareness of HFMD as a preceding infection for GBS, albeit rare. Despite motor deficits and the need for ventilation as predictors of poor prognosis, the early diagnosis and initiation of pharmacological therapy played a crucial role in the final outcome. The rehabilitation program, also instituted early in the course of treatment, was essential to the motor and functional recovery.
